# The Main Structural and Functional Characteristics of Photosystem-II-Enriched Membranes Isolated from Wild Type and *cia3* Mutant *Chlamydomonas reinhardtii*

**DOI:** 10.3390/life10050063

**Published:** 2020-05-14

**Authors:** Vasily V. Terentyev, Anna K. Shukshina, Aleksandr A. Ashikhmin, Konstantin G. Tikhonov, Alexandr V. Shitov

**Affiliations:** Institute of Basic Biological Problems, FRC PSCBR RAS, Pushchino 142290, Moscow Region, Russia; sshukshinka@gmail.com (A.K.S.); ashikhminaa@gmail.com (A.A.A.); ktikhonov@rambler.ru (K.G.T.); aleksshitow@rambler.ru (A.V.S.)

**Keywords:** *Chlamydomonas reinhardtii*, photosystem II, CAH3, chlorophyll, carotenoids, cytochrome b559, OJIP-kinetic

## Abstract

Photosystem II (PSII)-enriched membranes retain the original PSII architecture in contrast to PSII cores or PSII supercomplexes, which are usually isolated from *Chlamydomonas reinhardtii*. Here, we present data that fully characterize the structural and functional properties of PSII complexes in isolated PSII-enriched membranes from *C. reinhardtii*. The preparations were isolated from wild-type (WT) and CAH3-deficient mutant *cia3* as the influence of CAH3 on the PSII function was previously proposed. Based on the equal chlorophyll content, the PSII-enriched membranes from WT and *cia3* have the same amount of reaction centers (RCs), cytochrome b559, subunits of the water-oxidizing complex, Mn ions, and carotenes. They differ in the ratio of other carotenoids, the parts of low/intermediate redox forms of cytochrome b559, and the composition of outer light-harvesting complexes. The preparations had 40% more chlorophyll molecules per RC compared to higher plants. Functionally, PSII-enriched membranes from WT and *cia3* show the same photosynthetic activity at optimal pH 6.5. However, the preparations from *cia3* contained more closed RCs even at pH 6.5 and showed more pronounced suppression of PSII photosynthetic activity at shift pH up to 7.0, established in the lumen of dark-adapted cells. Nevertheless, the PSII photosynthetic capacities remained the same.

## 1. Introduction

Oxygenic photosynthesis takes place in cyanobacteria, algae, and higher plants. The unique reaction of photosynthetic water oxidation to molecular oxygen (O_2_) occurs within the water-oxidizing complex (WOC) located on the donor side of photosystem II (PSII) [1]. The WOC includes the Mn_4_CaO_5_ cluster as an active center and the extrinsic proteins of PSII [2,3]. It is thought that the evolutionary improvement of the Mn_4_CaO_5_ cluster, which may have originated from Mn-bicarbonate complexes in Archean (> 3 GYA) [4,5,6,7], was completed quickly because it has a highly conservative structure across all known oxygenic organisms. Interestingly, the same is observed for the intrinsic trans-membrane subunits of the PSII core complex, which are largely conserved from cyanobacteria to higher plants [2]. In contrast, the composition of extrinsic proteins surrounding the Mn_4_CaO_5_ cluster underwent large evolutionary changes [2,3,8]. The evolutionary development of the oxygenic photosynthetic apparatus most likely occurred along the line: cyanobacteria—green algae—higher plants; the last two are eukaryotic organisms. 

The thylakoids structure organization in the single chloroplast of the green algae cell is significantly different from the chloroplasts observed in the cells of higher plants [9]. The number of thylakoids per stack in the chloroplast of *Chlamydomonas reinhardtii* under normal light is usually near three to five, which is lower than in higher plants [9,10,11,12], and the length of stacked thylakoid membranes regions is much longer [9,10,11]. Additionally, the algal chloroplast contains a special organelle called pyrenoid, where RuBisCO is concentrated upon activation of the carbon-concentrating mechanism [11,13,14]. The pyrenoid is penetrated by unstacked thylakoids [9,10,15,16], that have minitubules [10].

Stacked thylakoid membranes mainly contain PSII complexes [12]. Therefore, the isolation of such particles allows the separation of the PSII-enriched regions of thylakoids from other parts containing photosystems I, b_6_f-cytochrome complexes, ATPases, etc., and protects the original PSII architecture. Thus, such preparations are commonly used for studying the structural and functional features of PSII at both optimal and stress conditions. However, PSII-enriched membranes of BBY-type [17,18] were previously isolated and thoroughly characterized, mostly from higher plants [17,18,19,20,21,22]. In *C. reinhardtii* PSII core complexes with the His-tag mutation as well as supercomplexes of PSII with outer light-harvesting complex (PSII–LHCII) have been isolated and well-characterized [1,23,24,25,26,27,28,29,30,31]. However, incorporation of His-tag into PSII core subunits may produce some harmful effects on the structural and, consequently, functional properties of PSII [26,30,31]. Simultaneously, the process of PSII–LHCII supercomplexes isolation often causes the loss of some extrinsic proteins [27,29,30,31]. PSII-enriched membranes of BBY-type have been isolated from *C. reinhardtii* only a few times by a team of co-workers to confirm the presence of lumenal carbonic anhydrase (CA) CAH3 near PSII [32,33,34]. However, the authors studied only some characteristics of the preparations, such as the O_2_ evolution rate, the CA activity, and the polypeptide composition, and the other part of the research was conducted using thylakoid membranes or whole cells [32,33]. Therefore, a detailed study of PSII-enriched membranes isolated from *C. reinhardtii* is required.

In general, the multi-subunit complexes of PSII in green algae and higher plants have a similar architecture, including the reaction center (RC) composed of D1 and D2 proteins and cytochrome b559 (Cyt b559); WOC located on the donor (lumenal) side of RC and composed of extrinsic proteins PsbO, PsbP, PsbQ, PsbR surrounding the Mn_4_CaO_5_ cluster; the inner (core) light-harvesting complex composed of CP43 and CP47 proteins; and the outer light-harvesting complex—LHCII [28,29]. At the same time, the proteins of the PSII core complex and the extrinsic proteins of PSII from green algae and higher plants have some differences in their structural properties. In particular, in *C. reinhardtii* the three extrinsic proteins, PsbO, PsbP, and PsbQ, directly bind to RC using independent binding sites [2,26,35]. In higher plants, only PsbO directly binds to RC, whereas PsbP and PsbQ are only associated with RC through interaction with PsbO [2,35,36]. In addition, the extrinsic proteins of PSII from green algae have lower molecular weights [26,35]. Algal LHCII differs in the protein number and organization of its trimers as compared to higher plants. The LHCII in higher plants is composed of three genes products (*Lhcb1-3*) organized as heterotrimers or homotrimers [37], while in green algae, LHCII consists of the products of nine genes (*LhcbM 1-9*) divided into four types [37,38,39]. Moreover, in *C. reinhardtii* four trimeric forms of LHCII were observed with different combinations of types I, II, and IV [39].

The presence of the highly active *α*-CA CAH3 near PSII in *C. reinhardtii* [33,34,40] is one more significant difference from higher plants. Regardless, the CA activity was also detected in PSII from higher plants [41,42,43,44,45], the carrier of that CA activity has not yet been identified. According to several reports, CAH3 is probably in close association with PSII where its CA activity can influence the WOC function [33,34,40]. Data have been published about the presence of CAH3 even in isolated PSII core complexes [32,33]. In addition, it was obtained previously, that the absence of the CAH3 subunit near WOC can cause significant changes not only in the functional properties of PSII but also in its structural characteristics [33]. It should be noted, that the participation of CAH3 in the carbon-concentrating mechanism was also proposed [15,16] because the presence of CAH3 in thylakoids penetrated of the pyrenoid was shown [15,16,32]. 

The photosynthetic apparatus of the *cia3* mutant of *C. reinhardtii* operates in the absence of CAH3. This occurs due to the presence of two changes in the amino acid composition in one of the transit peptides of CAH3 (the leucine pair substituted by arginine and methionine), which disrupts the transfer of the protein through the thylakoid membrane to the lumen [46]. The *cia3* cells require an elevated level of CO_2_ [46] after that they demonstrate an equal PSII photosynthetic activity compared to wild type (WT) cells [33,46].

Here, we present data that, for the first time, fully characterize the structural and functional properties of PSII multi-subunit pigment-protein complexes in isolated PSII-enriched membranes obtained from both WT and *cia3* cells of *C. reinhardtii*, which were grown under non-stress conditions. The results showed minor differences in the structural and functional properties of the preparations from both strains in contrast to previously published results [33]. The obvious differences in the PSII function were observed only after shifting of the preparations to non-optimal conditions for the WOC activity, which is consistent with our recent data [40]. The possible role of CAH3 in the differences between preparations from WT and *cia3* is discussed. 

## 2. Results

The pigment composition of PSII-enriched membranes isolated from both WT and *cia3* is shown in Table 1. We found no significant differences in chlorophyll (Chl) *a* and Chl *b* content between preparations isolated from the two strains. However, a small difference in the total carotenoid (Car) content was observed (*p* ~0.03), it was higher in WT. The Chl *a*/Chl *b* ratio, as well as the Chl/Car ratio, showed more statistically significant differences (*p* < 0.001 and *p* ~0.003, respectively) and both were higher in case of PSII-enriched membranes from *cia3*, despite the real differences being minor. 

**Figure 1:**
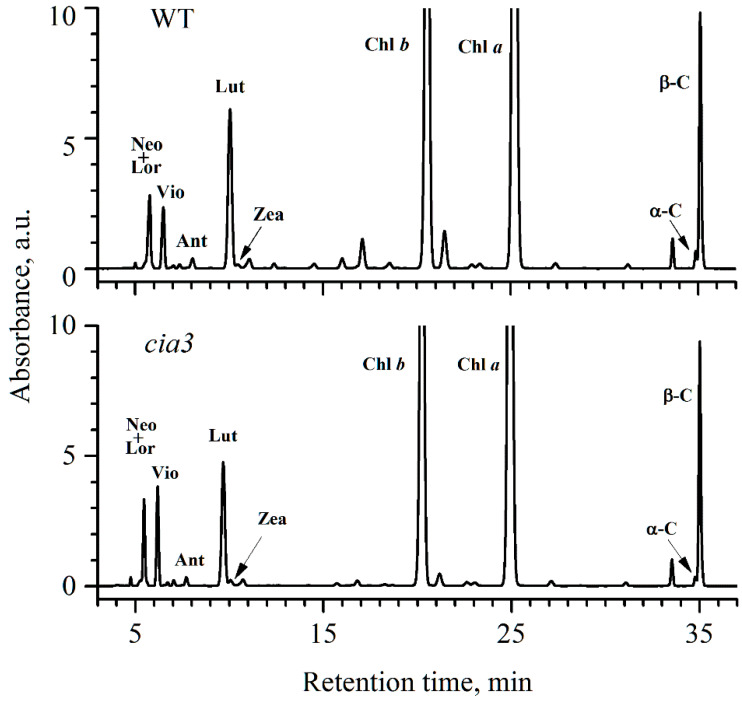
HPLC profiles of total pigments from acetone extracts of PSII-enriched membranes isolated from WT and *cia3*. The extracts were obtained from preparations based on the same concentration of Chl. For Car abbreviations, see the Table 2 caption.

The Car composition of PSII-enriched membranes was further analyzed using HPLC (Figure 1) and the obtained data are summarized in Table 2. The participants of both α-carotene (α-C) and β-carotene (β-C) branches of the Car biosynthetic pathway [47] were well resolved. It is known, that in *C. reinhardtii* cells α-C is usually present in very low amounts [47]. In PSII-enriched membranes from both WT and *cia3*, α-C was found in the lowest amount compared with other Car if the small amounts of antheraxanthin (Ant) and zeaxanthin (Zea) were not considered, which could be actively reversed to violaxanthin (Vio) by zeaxanthin epoxidase in the dark when the cells were prepared for the sample isolation procedure. Notably, the content of α-C in preparations from *cia3* was 1.5 times lower compared to the WT (Table 2). Lutein (Lut) is synthesized from α-C and, in contrast, is usually the most abundant Car in thylakoid membranes [48]. This is in agreement with the obtained results. As shown in Table 2, Lut was about 30%–37% of all Car in the PSII-enriched membranes. However, preparations from *cia3* contained less Lut compared to those from WT, which may be due to the initially lower amount of α-C in the case of *cia3*, as mentioned above (Table 2). Loroxanthin (Lor) is the last compound of the α-C branch. Unfortunately, it was impossible to separate the peaks of Lor and neoxanthin (Neo) (the last compound of the β-C branch) from each other in the obtained chromatograms (Figure 1). Nevertheless, the content of Neo + Lor was equal in PSII-enriched membranes from both WT and *cia3*. β-C was another Car widely presented in PSII-enriched membranes together with Lut (Table 2). The content of β-C in preparations was close to 36%–38% of all Car and it was slightly higher in *cia3*. In total, the β-C + Lut part was nearly 70% of all Car. 

Vio, Ant, and Zea, which are the participants of the β-C branch of the Car biosynthetic pathway, are thought to play the main role in the photoprotection of PSII, in contrast to Lut and Lor [48]. This is due to their involvement in the xanthophyll cycle, where under an excess of light, Vio is converted to Ant and then to Zea by violaxanthin de-epoxidase. Under normal light conditions or in the dark, Zea is converted to Ant and then to Vio by zeaxanthin epoxidase [47,48,49]. The fast accumulation of Zea (together with Ant) is critical for preventing PSII photoinhibition by reduction of singlet oxygen, dissipation of excess energy through non-photochemical quenching, and by other protective mechanisms [47,49].

In our PSII-enriched membranes, the Car of the xanthophyll cycle was mainly presented by Vio, which was ~1.5 times higher in preparations from *cia3* compared to those from WT (Table 2). Ant and Zea were found in preparations in low quantities (Table 2), and if the amount of Ant was close in both cases, Zea was higher in preparations from *cia3*. The total content of the Car of the xanthophyll cycle only was ~13.2% and ~17.9% for PSII-enriched membranes isolated from WT and *cia3*, respectively. However, the total Car content in WT preparation was slightly higher compared to that from *cia3* (Table 1), which may decrease the observed difference between the Car of the xanthophyll cycle.

**Figure 2:**
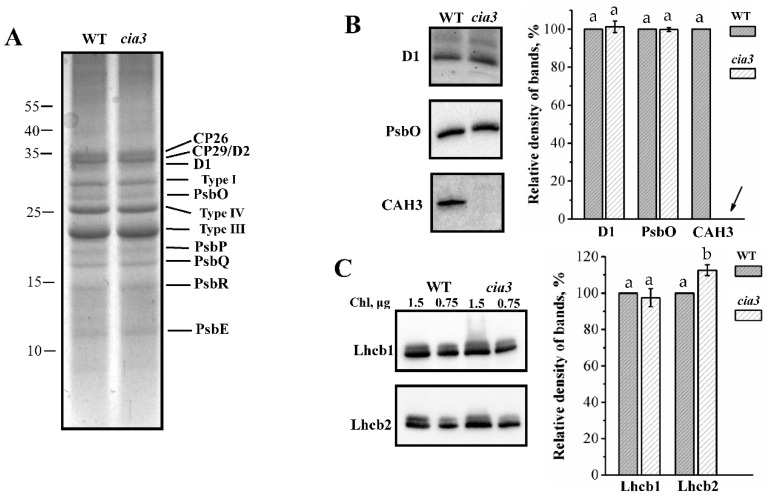
Results of SDS-PAGE (**A**) and Western blot (**B**,**C**) analysis of PSII-enriched membranes isolated from WT and *cia3*. In (**A**) bands corresponding to proteins of the PSII core complex and LHCII (Types I, III, and IV) are indicated. In (**B**,**C**) bands obtained by using primary antibodies against D1, PsbO, CAH3 and Lhcb1, Lhcb2 proteins are showed. Columns represent quantifications of the density of bands on the obtained blots from at least three separate experiments. The results were normalized to WT in each blot. The arrow indicates the absence of the signal on the blot. SD are shown as bars. The letters indicate a statistically significant difference between the values, *p* < 0.05. The samples were loaded on the gel in terms of the same amount of Chl.

The polypeptide composition of isolated PSII-enriched membranes was analyzed using SDS-PAGE (Figure 2A). Observed bands corresponded to the proteins of LHCII and PSII core complexes including WOC (PsbO, PsbP, PsbQ, and PsbR). The densities of the bands indicated the equal contents of the proteins in preparations from both WT and *cia3* in terms of the same amount of Chl. Additionally, PSII-enriched membranes were studied by Western blot using primary antibodies against D1 and PsbO proteins (Figure 2B), which are subunits of RC and WOC, respectively. Primary antibodies raised against *Arabidopsis* Lhcb1 and Lhcb2 proteins were used to study the composition of the outer antenna of PSII, LHCII (Figure 2C). The comparison of relative densities of the bands corresponding to D1 and PsbO proteins showed that PSII-enriched membranes isolated from both WT and *cia3* contained the same amount of PSII core complexes (or RCs) at an equal Chl concentration, in agreement with the SDS-PAGE results (Figure 2A). The same was observed for the band corresponding to the Lhcb1-like protein, which was obtained with a ~10% higher relative band density corresponding to the Lhcb2-like protein in PSII-membranes from *cia3* compared to that from WT (Figure 2C). This may reflect some differences in the composition of LHCII trimers in preparations from WT and *cia3*, which could explain the observed differences in the Chl *a*/Chl *b* ratio as well as in the Chl/Car ratio reported above (Table 1). The strong band of the CAH3 protein supported that this CA is widely presented in PSII-enriched membranes from *C. reinhardtii*. As expected, it was detected only in WT preparations (Figure 2B). 

**Figure 3:**
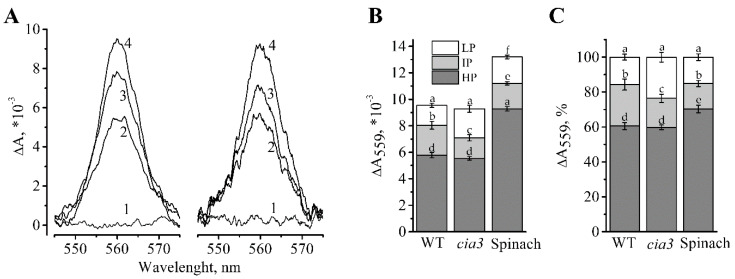
Difference reduced minus oxidized absorption spectra obtained for PSII-enriched membranes isolated from WT and *cia3* (**A**). The full oxidation of Cyt b559 was achieved by the addition of 50 μM potassium ferricyanide (1) and the subsequent reduction of the HP, IP, and LP forms were achieved by the stepwise addition of 5 mM hydroquinone (2), 5 mM sodium ascorbate (3) and sodium dithionite (4), respectively. The ratios of the different redox forms of Cyt b559 obtained from different spectra are presented on absolute (**B**) and normalized (**C**) scales for preparations from WT and *cia3* as well as from spinach from at least three separate experiments. SD are shown as bars. The letters indicate a statistically significant difference between the values, *p* < 0.05. The samples were used in terms of the same amount of Chl.

Another subunit of the PSII core complex is Cyt b559 [1]. The total Cyt b559 content in the preparations was determined by obtaining the full reduced minus oxidized difference spectra (Figure 3A) as we did previously [40]. The obtained results showed that PSII-enriched membranes isolated from both WT and *cia3* have the same amount of Cyt b559 (Figure 3A,B), i.e., the same amount of PSII core complexes, at equal Chl content. This supported the Western blot analysis findings described above (Figure 2B).

The ratio of different redox forms of Cyt b559 (low (LP), intermediate (IP), and high (HP) potential) were studied (Figure 3). It is known, that the part of the predominant HP form of Cyt b559 decreases under the presence of detergents, high or low pH, heating of samples, salt- or Tris-washing [50,51,52,53], etc. At the same time, the part of the LP form may increase upon the removal of some WOC proteins [22,52]. Therefore, the redox state of Cyt b559 may reflect the structural state of WOC, possibly through its connection with the PsbP protein [53]. As shown in Figure 3, PSII-enriched membranes from *cia3* showed a larger portion of the LP form compared to that in WT, which may reflect the presence of some WOC structural disturbance in the absence of the CAH3 subunit. The portion of the HP form was equal in preparations from both strains. For comparison, the total content of Cyt b559 and its different redox forms were determined in PSII-enriched membranes isolated from higher plants (spinach) (Figure 3B). 

The preparations from both WT and *cia3* contained by 36%–40% less Cyt b559 compared to preparations from spinach at an equal amount of Chl (Figure 3B). This was most likely due to the larger portion of LHCII per RC in PSII-enriched membranes isolated from algae. Data normalization allowed to compare the distribution of Cyt b559 redox forms in the preparations (Figure 3C). The PSII-enriched membranes from *C. reinhardtii* had ~10% less of the HP form compared to that from spinach, which could be a specific property of algal PSII. The portion of the LP form was the same in PSII from both WT *C. reinhardtii* and spinach, which was greater for PSII from *cia3* due to the reduction of the part of the IP form (Figure 3C), which can be induced by the absence of CAH3, as it was mentioned above.

Using the known reduced minus oxidized difference extinction coefficient of Cyt b559 for the α-band maximum at 559 nm, equal to 25.1 ± 0.5 mM^−1^ cm^−1^ [54], the Chl/RC ratio was calculated. This was ~300 Chl/RC for PSII-enriched membranes isolated from both strains *C. reinhardtii* and ~212 Chl/RC for preparations from spinach (Table 3). 

To study the state of the Mn_4_CaO_5_ cluster of the WOC of PSII in preparations isolated from both WT and *cia3*, we determined the total content of Mn and then calculated the Mn/RC ratio using the Chl/RC ratio obtained above. As shown in Table 3 the amount of Mn per RC was almost the same in PSII from both WT and *cia3*, however, the obtained values were lower than expected 4/1. The data showed that 12%–16% of RCs in PSII-enriched membranes did not have Mn_4_CaO_5_ clusters. This agrees with our results obtained previously from the rate of the 2,6-dichlorophenolindophenol (DCPIP) photoreduction by PSII from WT and *cia3* [40], which showed ~20% stimulation of the DCPIP photoreduction rate by an artificial electron donor (diphenylcarbazide) capable of direct electron donation to the primary electron donor of PSII independent of WOC (i.e., Mn_4_CaO_5_ cluster). As was proposed, this could reflect the fraction of PSII in preparations, which were in the assembling and/or degradation state and thus did not have Mn_4_CaO_5_ clusters.

**Figure 4:**
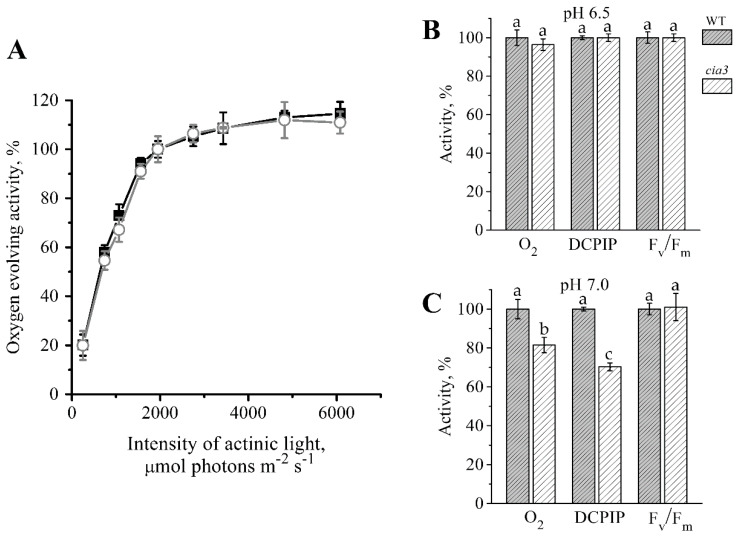
Dependence of the O_2_ evolution rate in PSII-enriched membranes from WT (■) and *cia3* (○) on the actinic light intensity (**A**). The O_2_-evolving activity at 1950 μmol photons m^−2^ s^−1^ was taken as 100%. Comparison of the O_2_ evolution (O_2_) and DCPIP photoreduction (DCPIP) rates as well as the value of the maximum quantum yield (F_v_/F_m_) obtained for PSII-enriched membranes isolated from WT (grey columns) and *cia3* (white columns) at pH 6.5 (**B**) and 7.0 (**C**). Each value is an average of 3–5 separate experiments with subsequent calculation of SD. The letters indicate a statistically significant difference between the values, *p* < 0.05. In each case, the value obtained in preparations from WT was taken as 100%. For more information, see the text.

Functionally PSII-enriched membranes isolated from both WT and *cia3* demonstrated equal photosynthetic activities at optimal pH 6.5. The dependence of the O_2_ evolution rate (i.e., the WOC function efficiency) on the actinic light intensity demonstrated the strong increase of this activity in preparations up to 1950 μmol photons m^−2^ s^−1^, which afterward reached a plateau. As shown in Figure 4A, the two obtained curves were similar. The maximum value (i.e., 100%; Figure 4B) of the O_2_ evolution rate obtained for preparations was ~280 μmol O_2_ (mg Chl h)^−1^. The DCPIP photoreduction reflecting the electron transport by PSII from water (Figure 4B) also showed an equal rate for PSII from both WT and *cia3* with a value of ~40 μmol DCPIP (mg Chl h)^−1^. Both values were consistent with our previously published data [40]. Additionally, the maximum quantum yield of PSII (F_v_/F_m_), which indicates the maximum photochemical efficiency of PSII [55,56], was estimated. The obtained F_v_/F_m_ values were the same for PSII from both WT and *cia3* (Figure 4B) and was near 0.65. Thus, the absence of the CAH3 protein does not influence these photosynthetic activities of PSII at optimal pH for its function. 

At the shift of pH up to 7.0, which can be reached in the thylakoid lumen in the dark or in the shadow [57,58,59,60] and suppresses the photosynthetic activity of PSII [24,40,61], significant differences in the detected activities of PSII from WT and *cia3* were observed. The activities were more inhibited in PSII from *cia3* lacking the CAH3 protein (Figure 4C). The rates of O_2_ evolution and DCPIP photoreduction decreased in PSII from WT to ~250 μmol O_2_ (mg Chl h)^−1^ and ~25 μmol DCPIP (mg Chl h)^-1^, respectively, while the value of F_v_/F_m_ changed to ~0.62. If we assumed the photosynthetic activities of PSII from WT to be 100%, the rates of O_2_ evolution and DCPIP photoreduction for PSII from *cia3* were suppressed by ~20% and ~30%, respectively. However, the value of F_v_/F_m_ remained the same for PSII from both WT and *cia3*. Based on the results of our previous study [40], these differences at pH 7.0 between PSII from WT and *cia3* may be due to the support of the WOC function by the CA activity of CAH3, which accelerates the removal of protons from the Mn_4_CaO_5_ cluster to the lumen in PSII from WT. At the same time, in PSII from *cia3* such CA activity is absent. At the same time, the CAH3 protein does not affect the maximum photochemical efficiency of PSII (Figure 4C). Nevertheless, the decrease in the O_2_ evolution and DCPIP photoreduction rates obtained in PSII from WT may be mainly explained by conformational changes of the WOC proteins induced by the pH shift; the same should occur in PSII from *cia3*. According to our previous data, these conformational changes are fully reversible up to pH 7.0 [40]. 

It was discussed previously [62,63,64], that the conformational changes of the PSII core proteins caused by the disturbance of the WOC native structure may affect the redox potential of the primary quinone acceptor, Q_A_, and consequently the Q_A_ and the secondary quinone acceptor (Q_B_) reduction/oxidation behavior on the PSII acceptor side. As mentioned above, the presence of a larger portion of the LP form of Cyt b559 in PSII-enriched membranes from *cia3* compared to WT (Figure 3) may be due to the presence of some structural disturbance of WOC in the absence of the CAH3 protein. Therefore, the conformational changes of the WOC proteins, which can be induced by a pH shift to 7.0 should be more pronounced in the case of PSII from *cia3*. To confirm that, the fast Chl *a* fluorescence induction kinetic, OJIP, which is defined by the redox state of Q_A_ [65,66,67], was obtained for PSII-enriched membranes isolated from both WT and *cia3* at pH 6.5 and 7.0. 

**Figure 5:**
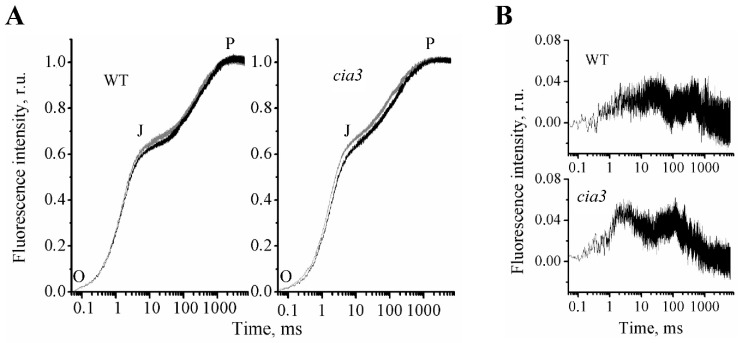
Fast Chl *a* fluorescence rise kinetics (OJP) of PSII-enriched membranes isolated from WT and *cia3* (**A**) at optimal pH equal 6.5 (black curve) and non-optimal pH equal 7.0 (grey curve) for PSII photosynthetic activity. Difference spectra (**B**) obtained by subtraction of the OJP curve at pH 6.5 from the OJP curve at pH 7.0 for preparations from WT (top) and *cia3* (bottom), respectively. The measurements were repeated at least three times with similar results.

The rise of fluorescence intensity in our preparations completely lost the I intermediate inflection (Figure 5A) usually observed in leaves, algal and cyanobacterial cells, chloroplasts, and thylakoid membranes at ~20 ms [67]. The J intermediate inflection was observed at ~5 ms. Therefore, the kinetics were characterized by a biphasic OJP pattern that fully agrees with the previously published data obtained for PSII-enriched membranes from spinach [20,21,68,69]. However, on the normalized scale, we observed that the fluorescence intensity at J in PSII-enriched membranes from *C. reinhardtii* was higher (~0.6; Figure 5A) compared to that obtained in PSII preparations from spinach (~0.42) [20,21]. P was reached at ~1 s in contrast to that usually obtained at ~200 ms for leaves, algal and cyanobacterial cells, chloroplasts, and even for thylakoids [67]. 

It is currently assumed that at P the full reduction of Q_A_ in all RCs is reached [65,66,67]. At the J inflection, the dominant fraction of RCs is mainly in the Q_A_^−^Q_B_ state (but some in the Q_A_Q_B_ or Q_A_^−^Q_B_^−^ state). The dip after J reflects the reoxidation of Q_A_^−^, probably by electron transfer to Q_B_, and at P, all RCs are in the Q_A_^−^Q_B_^2−^ state, i.e., RCs are closed [65,66,67].

In our measurements at pH 6.5 for PSII-enriched membranes isolated from both WT and *cia3*, the first O-J phase was the same, whereas the rise of the second J-P phase was more straightened for preparations from *cia3* (Figure 5A, black curves). To evaluate the observed differences, the 1 – Vj values and the complimentary area above the curve (area) were calculated from the obtained OJP kinetics. As follows from Table 4, fluorescence intensities at the J inflection were not statistically significantly different between preparations. However, the area decreased by 34% in PSII-enriched membranes from *cia3*. Thus the data indicated a somehow suppression of the electron transfer between Q_A_ and Q_B_ in PSII from *cia3* compared to that obtained for PSII from WT. 

These changes in the Q_A_ and Q_B_ reduction/oxidation behavior in PSII from *cia3*, as mentioned above, could be due to the presence of some conformational changes in the PSII core proteins, which can be induced by the absence of the CAH3 protein near WOC, resulting in some disturbance of the protein complex. The shift of pH up to 7.0, which should induce conformational changes of the proteins, led to the increase in the fluorescence intensity at the J intermediate inflection and, consequently, to the decrease in 1 – Vj and area values, even for PSII from WT (Figure 5A, Table 4), which supported the proposed role of conformational changes of the PSII core proteins in the suppression of the electron transfer between Q_A_ and Q_B_. The increase in the fluorescence intensity at the J intermediate inflection and the subsequent rise were significantly more pronounced in PSII from *cia3* (Figure 5, Table 4), suggesting the presence of stronger conformational changes in proteins of its WOC induced by pH in the absence of CAH3.

## 3. Discussion

The separation of PSII and other large multi-subunit complexes (photosystems I, b_6_f-cytochrome complexes, ATPases, etc.) of thylakoid membranes between stacked (mainly contain PSII) and unstacked regions [12] allows the isolation of PSII-enriched membranes with the original structure of PSII compared to PSII core complexes or PSII–LHCII supercomplexes, which are usually isolated from *C. reinhardtii*. From SDS-PAGE analysis (Figure 2A), we found that PSII-enriched membranes from both WT and *cia3* contained all WOC proteins (PsbO, PsbP, PsbQ, and PsbR) in equal contents. The well-defined bands of LHCII proteins (Types I, III, and IV) were also observed in equal contents. This conclusion was supported by the Western blot analysis, which also showed the same contents of proteins of the PSII core complex D1 (RC) and PsbO (WOC) in the preparations (Figure 2B). The determination of the total content of Cyt b559, which is in a 1:1 ratio with RC in PSII [1], showed the same result: an equal content of PSII core complexes per Chl in WT and *cia3* (Figure 3). However, this finding differs from the data obtained previously, where *cia3* had significantly more PSII proteins in thylakoid membranes compared to WT [33]. The most likely explanation of the observed contradiction is the different growing light conditions. According to previously published data, an increase in light intensity from ~50 up to ~500 μmol photons m^−2^ s^−1^ induces changes in the photosynthetic apparatus of *C. reinhardtii*, including a decrease of total Chl as well as photosystem I content and an increase in the Chl *a*/Chl *b* ratio due to the reduction of LHCIIs [11]. In addition, the suppression of the photosynthetic activity of *cia3* cells was observed in experiments at light intensities higher than 300 μmol photons m^−2^ s^−1^ in contrast to WT cells [70]. In the previous work [33], algae were grown at a light intensity of ~150 μmol photons m^−2^ s^−1^, which probably had an insignificant influence on the photosynthetic apparatus from WT *C. reinhardtii*, whereas, for *cia3*, this corresponded to high light conditions. In turn, we used a light intensity of ~90 μmol photons m^−2^ s^−1^ at the surface of the bottles, which was additionally decreased due to passing through the glass and dense culture. This light intensity was not enough to induce the changes observed in the previous work [33], corresponding to non-stress conditions.

In PSII-enriched membranes, we also analyzed the bands of proteins recognized by antibodies raised against the higher plant proteins Lhcb1 and Lhcb2 (Figure 2C), which are the major components of LHCII in higher plants [71,72]. A similar approach was used previously for studying the LHCII composition of PSII from green algae [73,74,75]. For our preparations, the intensity of the band corresponding to the Lhcb1-like protein was equal for PSII from both WT and *cia3*, whereas the band corresponding to the Lhcb2-like protein was ~10% higher for PSII-enriched membranes from *cia3* (Figure 2b). These results indicated some differences in the LHCII trimers composition in the outer antenna in preparations from WT and *cia3*. This suggestion is supported in particular by the Car composition data. Obvious but minor differences in Car concentrations were detected (Table 1), characterized by differences in their relative distributions for both WT and *cia3* (Figure 1, Table 2). Despite the fact, that the sum of Car belonging to LHCII (Neo, Lor, Vio, Ant, Zea, Lut [29,49,76]) was almost the same per Chl (~61%), the Lut content was higher in PSII from WT, whereas the content of xanthophyll cycle pigments (Vio, Ant, Zea) was higher in PSII from *cia3* (Table 2). Thus, some difference probably exists in the combination of LhcbM subunits with varied Car compositions, which form the LHCII trimers in WT and *cia3*.

We found no significant difference in the Chl *a* and Chl *b* content for PSII-enriched membranes isolated from both WT and *cia3* (Table 1). This finding contrasts with previously published data [33] and can be explained by the high light stress of *cia3* presented in that work, as discussed above. Using the known reduced minus oxidized difference extinction coefficient of Cyt b559, we were able to calculate the Chl/RC ratio in our preparations from WT and *cia3*. This ratio was near 300 in both cases and higher compared to PSII-enriched membranes isolated from spinach by ~40% (Table 3). Based on the determined Chl/RC ratio, we calculated the content of Mn per RC, which was also the same for preparations from both WT and *cia3* (Table 3). However, it was surprisingly lower than 4 Mn per RC due to about 12%–16% of RCs not having the Mn_4_CaO_5_ cluster, possibly because they belong to the assembly and/or degradation PSII complexes, which is consistent with the data reported in our recent work [40]. Previously, the Mn/RC ratio was estimated to be 4/1 for thylakoids from *C. reinhardtii* grown in low light but this was based on a Chl/RC ratio of 250 [25], which is usually used for PSII-enriched membranes from higher plants [18]. In another work, the authors calculated that 4 Mn in PSII membranes from *C. reinhardtii* are equivalent to 276 Chl [77]. As we showed here, the preparations from *C. reinhardtii* have more Chl molecules per RC (Table 3). Thus, if we were to recalculate the results in [25,77] using the Chl/RC ratio from the present work, the results would agree with ours. 

Another observation was the similar contents of carotenes, which was also related to the equal content of RCs in terms of Chl in our PSII-enriched membranes from both WT and *cia3*. It is known, that the main carotene of thylakoid membranes is β-C, which is localized in PSII complex within RC [1,78], whereas α-C, which usually presents in the lowest amount in thylakoid membranes, binds to the same sites in RC, replacing the β-C [78,79]. Thus, the total amount of β-C plus α-C should reflect the amount of total RCs. In our PSII-enriched membranes, this sum for WT and *cia3* was almost the same (38%–39%). 

At the same time, the obtained data probably reflected the presence of some WOC disturbance induced by the absence of CAH3. As reported previously [62,63,64], the disturbance of WOC on the PSII donor side may affect the Q_A_ redox properties on the PSII acceptor side through the induced conformational changes of PSII core proteins. Therefore, we propose that the different architecture of algal WOC compared to that of higher plants [2,26,35] could influence the Q_A_ and Q_B_ reduction/oxidation behavior. Indeed, the obtained OJP kinetics of preparations from *C. reinhardtii* had a relatively higher J inflection level compared with the value published for spinach [20,21]. This reflected the presence of a larger portion of reduced Q_A_, which could be due to the decrease of the electron transfer from Q_A_ to Q_B_. At the same time, the OJP kinetics of PSII from *cia3* displayed a more straightened J-P phase compared with WT (Figure 5, Table 4), which can be explained by the additional suppression of the electron transfer between Q_A_ and Q_B_. Most probably, this occurs through some disturbance in the WOC native structure induced by the absence of CAH3 protein, which can also follow from the increased portions of the LP form of Cyt559 in PSII from *cia3* (Figure 3). Experiments at increased pH (Figure 5, grey curves), which should induce conformational changes of proteins, showed the increase of the J inflection level and the decrease of the area above the OJP curve for PSII from WT (Table 4). However, more dramatic changes in OJP kinetic shape were observed for PSII from *cia3*, supporting the suggestion about the initial presence of disturbance of the WOC structure and, consequently, conformational changes in the PSII core proteins at pH 6.5 in the absence of CAH3. 

Nevertheless, the photosynthetic capacity of PSII from both WT and *cia3* defined as the maximum quantum yield (F_v_/F_m_) remained the same even at pH 7.0 (Figure 4C). Therefore, the above-described differences probably reflected some changes in the WOC structure induced by the absence of CAH3 near PSII that do not affect the long-term operation of PSII in cells grown under non-stress conditions.

## 4. Materials and Methods

### 4.1. Strains and Growth Conditions

The cell wall-less mutant CC-503 cw92 mt + of *C. reinhardtii*, which is used as the standard WT, was purchased from the Chlamydomonas Resource Center, University of Minnesota, USA. The cell wall-less and CAH3-deficient double mutant *cia3* [32,33,34] was kindly provided by G. Samuelsson (University of Umeå, Sweden). Both strains were grown photoautotrophically in minimal medium at 25 °C under aeration with air enriched with 5% CO_2_ as described previously [40]. Continuous illumination was provided from cool white fluorescent lamps at 90 μmol photons m^−2^ s^−1^. Cells were harvested in the mid-exponential phase of growth.

### 4.2. Isolation of PSII-Enriched Membranes

Cells of *C. reinhardtii* were collected by centrifugation at 3000 *g*, 10 min, and washed with chilled buffer A (50 mM Hepes-NaOH (pH 7.8), 350 mM NaCl, 2 mM EDTA). Before disruption 1 mM sodium ascorbate was added to the buffer. Cells were disrupted by passing through a precooled French pressure cell (Thermo Scientific, Waltham, MA, USA) at 28 MPa (4000 Psi). Whole cells and large debris were harvested with a low-speed spin (500 *g*, 5 min), and then membranes were pelleted at 7000 *g*, 30 min. The pellet was resuspended in chilled buffer B (50 mM Hepes-NaOH (pH 7.8), 3 mM NaCl, 5 mM MgCl_2_, 2 mM EDTA, 200 mM sucrose, 1 mM sodium ascorbate) and precipitated at 7000 *g*, 30 min. The upper green layer of the pellet, which contained primarily thylakoid membranes, was carefully resuspended by paintbrush in chilled buffer C (20 mM MES-NaOH (pH 6.5), 15 mM NaCl, 5 mM MgCl_2_, 300 mM sucrose) to a Chl concentration of 3–4 mg mL^−1^. For the PSII-enriched membrane fragments (BBY-type [18]) isolation, 20% (w/w) Triton X-100 solution was added to the thylakoid membranes (Triton X-100 to Chl ratio was as 20:1 (w/w) and the final concentration of Chl was 2 mg mL^−1^), and the suspension was incubated for 20 min on ice under gentle stirring in the dark. PSII-enriched membranes were harvested by centrifugation at 40,000 *g*, 35 min, and washed thrice in the buffer C. Finally, the pellet was resuspended in the buffer C containing 10% glycerol to a Chl concentration of 2–3 mg mL^−1^, homogenized and frozen at −80 °C. All steps were carried out at 4 °C and green dim light.

### 4.3. Pigment Concentrations

The concentration of total Chl, as well as the distribution between a and b forms and the concentration of total Car, were determined spectroscopically after extraction in 80% acetone [80,81].

### 4.4. Carotenoids Composition

For separation of Car, 9 ml of acetone:methanol mixture (7:2, *v/v*) were added to 2 mL of a sample extract in 80% acetone (at 10 μg mL^−1^ of Chl) under continuous stirring, after that 4 ml of petroleum and 25 mL of water were successively added and the suspension mixed again. The pigments localized in the upper layer of the mixture were pipetted, transferred into a heparin vial and dried under argon flow. The obtained film of pigments was diluted in the acetone:methanol mixture (7:2, *v/v*) and 20 µL of the extract was used for HPLC analysis. An HPLC system consisted of (1) a pump LC-10ADVP with a module FCV-10ALVP, (2) a detector with diode matrix SPD-M20A, and (3) a thermostat CTO-20AC (Shimadzu, Japan). The separation of the Car was performed on a 4.6 × 250 mm reversed-phase column (Agilent Zorbax SB-C18, Agilent, Santa Clara, CA, USA) at 22 °C. The following solvent gradient was used at a flow rate 1 mL min^−1^: the solution A (acetonitrile:ethyl acetate:water, 69:23:8, *v/v*) after 5 min was replaced with a linear gradient (0%–25%) of the solution B (ethyl acetate) for 20 min, and then the solution B was gradually increased to 100% for 20 min. At the end of the analysis, 100% of solution B was passed through the column for 3 min. The Car were identified by their retention time and absorption spectra. The quantification of each Car was performed by comparing its peak area in the region of 360–800 nm to the sum of all Car peaks taken as 100% and was calculated with the LC-solution program (Shimadzu, Kyoto, Japan).

### 4.5. SDS-PAGE and Western Blot Analysis

Proteins separation of PSII-enriched membranes isolated from WT and *cia3* was carried out by electrophoresis under denaturing conditions in a 16% polyacrylamide gel [82] in Mini-PROTEAN 3 Cell (BioRad). The samples were loaded on a gel at an equal amount of Chl content equal to 1.5 μg per track unless otherwise indicated. After electrophoresis, the proteins were transferred onto a nitrocellulose membrane (Amersham, Protran, 0.45 μm NC) using the Mini Trans-Blot Cell wet blotting system (BioRad). The membrane was incubated overnight at 4 °C with anti-rabbit primary antibodies against D1, PsbO, Lhcb1, Lhcb2, and CAH3 proteins produced by Agrisera (Sweden) (AS11 1786, AS06 142-33, AS01 004, AS01 003 and AS05 073, respectively). Donkey anti-rabbit antibodies labeled with horseradish peroxidase (GE Healthcare) were used as secondary antibodies in a dilution of 1:5000. The antibody-antigen conjugates were detected by a Pierce ECL Plus Western Blotting kit (Thermo scientific) and the gel documentation system ChemiDoc (BioRad). Quantification of bands on the blots was performed by ImageJ software (version 1.53a, National Institutes of Health, Bethesda, MD, USA).

### 4.6. Analysis of Redox Forms of Cytochrome b559

Determination of the different redox forms of Cyt b559 in PSII-enriched membranes was performed by measurement of absorption changes at 559 nm (ΔA_559_) from reduced minus oxidized difference spectra in the region of 540–580 nm in the presence of different redox agents as described previously [22,40]. Before measurements the samples were diluted in a buffer, containing 20 mM MES-NaOH (pH 6.5), 35 mM NaCl, 400 mM sucrose, to Chl concentration of 100 μg mL^−1^. Complete oxidation of sample suspension was achieved by the addition of 50 μM potassium ferricyanide, and the reduction was performed by stepwise addition of 5 mM hydroquinone, 5 mM sodium ascorbate and a few grains of dithionite for detection of low (LP), intermediate (IP) and high (HP) potential forms of Cyt b559, respectively, with the recording of the different absorption spectrum in each step. For determination of the total content of Cyt b559 in PSII-enriched membranes the dithionite minus potassium ferricyanide different absorption spectrum was used. 

### 4.7. Determination of Metal Content

Mn content in PSII-enriched membranes suspensions (at 100 μg Chl mL^−1^ in 1 M HNO_3_) was determined by using a flame atomic absorption spectrometer Kvant-2A (Cortec, Moscow, Russia) at 279.5 nm.

### 4.8. O_2_-Evolving Activity Measurements

The rate of photosynthetic oxygen evolution was measured at 25 °C with a Clarke-type electrode, in a 1-mL cell (Hansatech Instruments Ltd., Norfolk, UK) as it was performed previously [22,40,43] at 1950 μmol photons m^−2^ s^−1^ of red light (λ > 600 nm). Measurements were carried out in medium, containing either 20 mM MES-NaOH (pH 6.5) or 20 mM MOPS-NaOH (pH 7.0) and 35 mM NaCl, 400 mM sucrose. 1 mM potassium ferricyanide and 0.2 mM 2,6-dichloro-p-benzoquinone were used as electron acceptors [40]. The Chl concentration during measurements was 10 μg mL^−1^. The study of light dependency was performed at pH 6.5 with the addition of a new sample for each point on curves as well as for each repetition.

### 4.9. Electron Transfer Rate Measurements

Photoinduced electron transfer from water to the electron acceptor 2,6-dichlorophenolindophenol (DCPIP) was measured spectroscopically by the decrease in photoinduced absorption at 600 nm as a result of DCPIP reduction. The assay was performed in the same medium as it was done in the measurements of oxygen evolution at Chl concentration of 10 μg ml^−1^. Different extinction coefficients of 50 μmol DCPIP were used for pH 6.5 and 7.0 due to the dependence of DCPIP properties on pH [40,83].

### 4.10. Chlorophyll Fluorescence Measurements

The values of the maximum quantum yield of PSII calculated as ratio variable/maximal fluorescence (F_v_/F_m_) were detected using an XE-PAM fluorometer (Walz, Germany). The fast Chl *a* fluorescence rise kinetics (OJIP) were measured using a Multi-Color PAM fluorometer (Walz, Germany) with the lowest intensity of measuring light (intensity 1) and the highest intensity of red (~650 nm) actinic light (intensity 20). Fluorescence intensity at time t (F(t)) was normalized as V(t) = (F(t) – F_0_)/(F_m_ – F_0_), where F_0_ and F_m_ are the lowest fluorescence intensity before the actinic light was switched on, i.e., O (at ~0.05 ms) and the maximum of fluorescence intensity obtained under actinic light, i.e., P, respectively. The 1 – Vj parameter was calculated as 1 – (F_j_ – F_0_)/(F_m_ – F_0_), where F_j_ is fluorescence intensity at the J inflection (at 5 ms). All measurements were performed in 10 mm × 10 mm quartz cuvette (Hellma Analytics) at room temperature in the same medium as it was used in the measurements of oxygen evolution and DCPIP photoreduction and at Chl concentration of 10 μg ml^−1^. Preparations were adapted to the medium for 3 min in the dark with average stirring.

### 4.11. Statistical Analysis

Statistical analysis was performed using standard algorithms of OriginPro 2016 (OriginLab, Northampton, USA). The data are presented as the mean ± SD. Differences with *p*-values < 0.05 were considered statistically significant. 

## Figures and Tables

**Table 1:** Pigment composition of PSII-enriched membranes in terms of 1 mg of total Chl. Each value is an average of at least 3 separate experiments with a calculation of SD.

	Chl *a*, μg	Chl *b*, μg	Chl *a*/Chl *b*	Car, μg	Chl/Car
WT	714.3 ± 7.2 ^a^	285.6 ± 2.7 ^b^	2.5 ± 0.01 ^c^	208.7 ± 1.1 ^e^	3.4 ± 0.02 ^g^
*cia3*	722.6 ± 11.7 ^a^	276.7 ± 4.8 ^b^	2.6 ± 0.01 ^d^	201.9 ± 3.3 ^f^	3.6 ± 0.01 ^h^

The letters indicate a statistically significant difference between the values, *p* < 0.05.

**Table 2:** Distribution of different Car by % in PSII-enriched membranes. Neo—neoxanthin, Lor—loroxanthin, Vio—violaxanthin, Ant—antheraxanthin, Lut—lutein, Zea—zeaxanthin, α-C—α-carotene, β-C—β-carotene. Each value is an average of at least 3 separate experiments with the calculation of SD.

	Neo + Lor	Vio	Ant	Lut	Zea	α-C	β-C
WT	11.5 ± 0.8 ^a^	10.1 ± 0.2 ^b^	2.1 ± 0.06 ^d^	37.6 ± 0.8 ^e^	1.0 ± 0.06 ^g^	2.3 ± 0.06 ^i^	35.5 ± 1.0 ^k^
*cia3*	12.3 ± 0.8 ^a^	14.7 ± 1.1 ^c^	1.9 ± 0.11 ^d^	30.1 ± 0.6 ^f^	1.3 ± 0.07 ^h^	1.4 ± 0.05 ^j^	37.7 ± 1.4 ^e,k^

The letters indicate a statistically significant difference between the values, *p* < 0.05.

**Table 3:** Calculated ratios of Chl and Mn to RC. The RCs content in PSII-enriched membranes was calculated based on the total Cyt b559 content obtained by its full reduced minus oxidized difference spectra (Figure 3A). For determination of Mn content 10–12 separate measurements were made.

	WT	*cia3*	spinach
Chl/RC	295 ± 2 ^a^	303 ± 8 ^a^	212 ± 2 ^b^
Mn/RC	3.52 ± 0.29 ^c^	3.36 ± 0.20 ^c^	–

The letters indicate a statistically significant difference between the values, *p* < 0.05.

**Table 4:** Parameters calculated from OJP curves (Figure 5A) and showed the efficiency of the electron transfer further than the primary quinone acceptor, Q_A_^−^. The decrease of values in both cases reflects, accordingly, the decrease of the efficiency [66]. Area–total complementary area between the OJP curve and F = F_m_.

	1 – Vj		Area, %	
	pH 6.5	pH 7.0	pH 6.5	pH 7.0
**WT**	0.420 ± 0.009 ^a^	0.399 ± 0.007 ^b^	100 ± 2 ^*^	90.7 ± 4
*cia3*	0.409 ± 0.007 ^ab^	0.376 ± 0.003 ^c^	66.1 ± 3	49.5 ± 2

The letters indicate a statistically significant difference between the values, *p* < 0.05. *This value was taken as 100%.

## Abbreviations

PSII	Photosystem II
RC	Reaction center
WOC	Water-oxidizing complex
LHCII	Light-harvesting complex II
WT	Wild type
CA	Carbonic anhydrase
Chl	chlorophyll
Car	carotenoids
α-C	α-carotene
β-C	β-carotene
Neo	Neoxanthin
Lor	Loroxanthin
Vio	Violaxanthin
Ant	Antheraxanthin
Lut	Lutein
Zea	Zeaxanthin
Cyt b559	cytochrome b559
LP, IP, HP	low-, intermediate-, high- potential forms of Cyt b559
DCPIP	2,6-dichlorophenolindophenol
F_v_/F_m_	maximum quantum yield of PSII
